# An improved tensile deformation model for *in-situ* dendrite/metallic glass matrix composites

**DOI:** 10.1038/srep13964

**Published:** 2015-09-10

**Authors:** X. H. Sun, J. W. Qiao, Z. M. Jiao, Z. H. Wang, H. J. Yang, B. S. Xu

**Affiliations:** 1 Laboratory of Applied Physics and Mechanics of Advanced Materials, College of Materials Science and Engineering, Taiyuan University of Technology, Taiyuan 030024, China; 2 Key Laboratory of Interface Science and Engineering in Advanced Materials, Ministry of Education, Taiyuan University of Technology, Taiyuan 030024, China; 3 Institute of Applied Mechanics and Biomedical Engineering, Taiyuan University of Technology, Taiyuan 030024, China

## Abstract

With regard to previous tensile deformation models simulating the tensile behavior of *in-situ* dendrite-reinforced metallic glass matrix composites (MGMCs) [Qiao *et al.*, Acta Mater. 59 (2011) 4126; Sci. Rep. 3 (2013) 2816], some parameters, such as yielding strength of the dendrites and glass matrix, and the strain-hardening exponent of the dendrites, are estimated based on literatures. Here, Ti_48_Zr_18_V_12_Cu_5_Be_17_ MGMCs are investigated in order to improve the tensile deformation model and reveal the tensile deformation mechanisms. The tensile behavior of dendrites is obtained experimentally combining nano-indentation measurements and finite-element-method analysis for the first time, and those of the glass matrix and composites are obtained by tension. Besides, the tensile behavior of the MGMCs is divided into four stages: (1) elastic-elastic, (2) elastic-plastic, (3) plastic-plastic (work-hardening), and (4) plastic-plastic (softening). The respective constitutive relationships at different deformation stages are quantified. The calculated results coincide well with the experimental results. Thus, the improved model can be applied to clarify and predict the tensile behavior of the MGMCs.

Bulk metallic glasses (BMGs) exhibit superior mechanical performances at ambient temperature, such as high strengths, large elastic limits, and excellent corrosion and wear resistance^1^,^2^. Nevertheless, their room-temperature poor ductility upon loading remains the main obstacle to their usage in structural applications. Lack of pronounced macroscopic plasticity in BMGs is associated with highly-localized shear banding, and very limit plasticity is accumulated in the narrow shear bands, exhibiting strain softening caused by adiabatic shearing^3^. Research on the plasticity of BMGs aims at overcoming the drawback of lack of macroscopic plasticity has resulted in the development of a series of *in-situ* dendrite-reinforced metallic glass matrix composites (MGMCs)^4^,^5^,^6^,^7^. The microstructure of the *in-situ* MGMCs consists of ductile crystalline phases, embedded in a fully glass matrix. Shear banding is a ubiquitous plastic-deformation mode in the glass matrix^8^,^9^. Shear bands within the glass matrix will inevitably pass through the dendrites, and dendrites are under shear deformation^9^. Undoubtedly, the propagation of shear bands could be hampered by the ductile dendrites, and, as a consequence, a distinguishing plasticity can be eventually achieved^4^,^5^,^6^,^7^.

However, it should be noted that most of the developed *in-situ* dendrite-reinforced MGMCs exhibit softening upon tension rather than work hardening^4^,^5^,^6^,^7^, and it is due to that the tensile and the compressive behaviors are very different^10^,^11^. In order well to understand the tensile mechanisms comprehensively, not only the tensile behaviors of the glass matrix, dendrites, and corresponding composites, but also the constitutive relationships at different deformation stages are badly needed to reveal tensile deformation mechanisms. Qiao *et al.*^9^,^12^ have previously proposed the deformation mechanisms of *in-situ* dendrite-reinforced MGMCs. However, some parameters in previous studies^9^,^12^, such as the yielding stresses of the dendrites and glass matrix, and the strain-hardening exponent of the dendrites, are estimated based on literatures. In this study, a simple and effective way is employed to calculate these parameters. Dao *et al.*^13^ has proposed an approach to extract the plastic properties or constitutive relations in metals from nano-indentation tests, combining the finite-element-method (FEM) analysis, by which the stress-strain curve of dendrites can be easily obtained. Based on the stress-strain curves of the glass matrix, dendrites, and composites, the constitutive relationships are established to explain tensile behavior, and the theoretical calculations agree well with the experimental results. The proposed improved model for *in-situ* MCMCs upon room-temperature tension may be a guideline to design MGMCs with large tensile ductility.

## Results

### Microstructures

**Figure 1:**
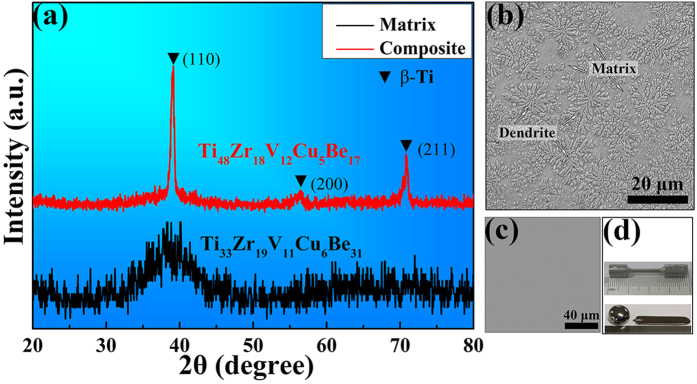
The XRD patterns of the composites and the glass matrix in (**a**), the SEM image of the as-cast composites in (**b**) and the glass matrix alloy in (**c**). The tensile sample of the present composites in the upper of (**d**) and the cast ingots of the glass matrix alloy in the lower of (**d**).

Figure 1(a) shows the XRD patterns of the as-cast composites in red and the matrix alloy in black with the nominal compositions of Ti_48_Zr_18_V_12_Cu_5_Be_17_ and Ti_33_Zr_19_V_11_Cu_6_Be_31_, respectively. The XRD pattern of the as-cast composites displays sharp diffraction peaks of body-centered-cubic dendritic phase (*β*-Ti) adding to the broad diffuse scattering maxima of the glass phase, indicating the presence of the crystalline phase in the glass matrix. The XRD result of the matrix alloy indicates that a typical broad hump is observed with no visible crystalline diffraction peaks, indicating that the as-cast matrix alloy is fully amorphous. Figure 1(b) displays the microstructure of the as-cast MGMCs, and the microstructure exhibits a typical dual-phase morphology. The coarsen dendrites are homogeneously dispersed in the featureless glassy matrix. The average diameter of the dendritic arms is 2 μm, and the volume fraction of dendrites is ~42%. Figure 1(c) shows the microstructure of the as-cast matrix alloy. There is no contrast from the SEM image, suggesting the amorphous structure of the matrix alloy, in accordance with the XRD results.

### Nano-indentation and FEM analysis

As one of phases in the present MGMCs, the dendrites play an important role in gaining the tensile ductility of the MGMCs^4^,^5^,^6^,^7^. The propagation of shear bands could be hampered by the dendrites, and, therefore, a distinguishing plasticity can be eventually achieved^4^,^5^,^6^,^7^. In the previous models^9^,^12^, the dendrites undergo both the elastic and plastic deformation behaviors. Some parameters of established tensile deformation models for dendrites, such as the yielding stresses and strain-hardening exponent are estimated based on literatures. In order to accurately establish tensile deformation models for dendrites in this study, the method proposed by Dao *et al.*^13^ has been employed. The approach can experimentally extract the plastic properties or constitutive relations in metals from nano-indentation tests, combining the finite-element simulation, by which the stress-strain curve of dendrites can be easily obtained.

During nano-indentation tests, a sharply-rigid indenter normally penetrates into dendrites, where the indentation load, *P*, and displacement, *h*, are continuously registered in one loading–unloading cycle. Upon loading, the Kick’s Law^13^ can be described as follows:


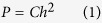


where *C* is the loading curvature, and it can be obtained by the curve fitting.

The maximum indentation depth, *h*_max_, occurs at the maximum load, *P*_max_. The initial unloading slope (contact stiffness) can be described as follows^13^:


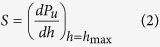


where *P*_u_ is the unloading load.

**Figure 2:**
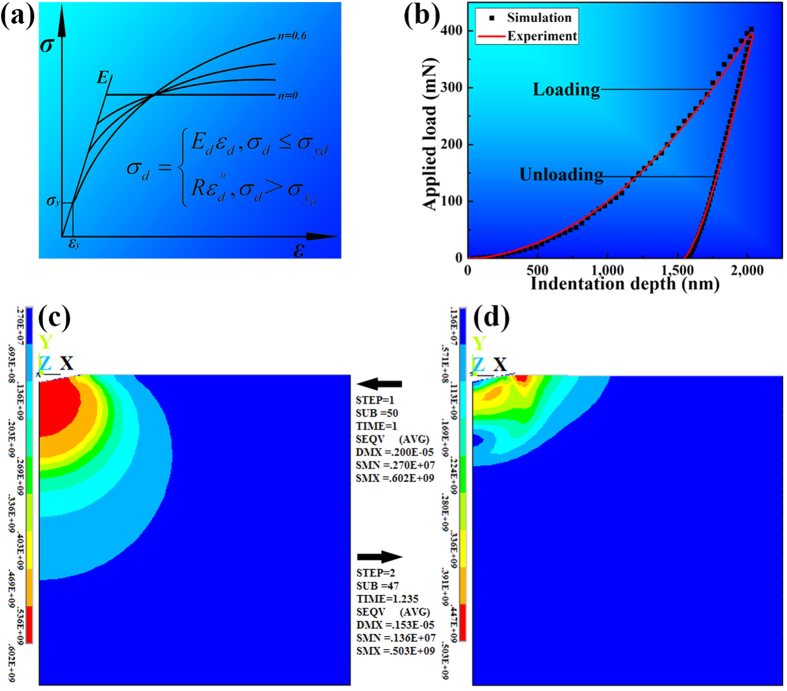
A schematic diagram of the tensile behavior of dendrites in (**a**), the simulative and experimental loading and unloading curves for the dendrites in (**b**), and the contour maps of the stress distribution at the maximum depth in (**c**), and the residual depth in (**d**).

An axisymmetric finite-element model of the semi-infinite space is developed to simulate nano-indentation tests. The pyramid shaped indenter is treated the same as the conical indenter with a cone angle of 70.3°, providing the same area to depth relationship. Surface-to-surface contact elements are applied to the exposed surfaces. The friction coefficient between the tip and the specimen surface is assumed to be 0.16^14^. Since the largest fracture strain is ~15.5% in the Ti-Zr-V-Cu-Be alloy systems^9^, it is assumed that the fracture strain is 15% for the dendrites in the current composites. The commercial finite-element package ANSYS v.10.0 is applied for the static analysis. The plastic behavior of the dendrites can be closely approxiated by a power-law description^13^, as shown schematically in Fig. 2(a). The constitutive equation of dendrites is expressed as follows:


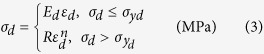


where *σ*_*d*_, *E*_*d*_, *ε*_*d*_, *σ*_*yd*_, *R*, *n*, and *ε*_*pd*_ are the stress, Young’s modulus, total effective strain, yielding stress, strength coefficient, work-hardening exponent, and plastic strain of the dendrites, respectively. To complete the constitutive description, the Poisson’s ratio of the dendrites, *υ*_*d*_, is assumed to be 0.33^15^,^16^,^17^,^18^. With the above assumptions and definitions, the constitutive relation of dendrites is fully determined by the following parameters *E*, *σ*_*y*_, and *n*.

The simulated stress–strain curve of dendrites should be divided into two stages: elastic and plastic stages. Combining the elastic and plastic deformation behaviors, the tensile behavior of the dendrites is characterized. At the elastic stage, the indentation loading curves of materials with the same *E* are almost independence of the strain-hardening exponent, as shown in Fig. 2(a). Adopting the special case of *n* = 0, the elastic modulus and yielding stress can be determined by continuous fitting to the measured applied load–indentation depth relations until the satisfied agreement between them is reached^8^. When *E* = 50 GPa, *σ*_y_ =  = 500 MPa, the simulated and experimental results have a good agreement upon loading, as shown in Fig. 2(b), and the yielding strain is 0.01, which is very closed to the reported Ti-alloy yielding strain with a value of 0.007 ~ 0.01^15^.

At the plastic stage, the modificatory simulations are used to acquire the strain-hardening exponent. Finite-element simulation begins with the strain-hardening exponent *n* = 0.07^9^ within the range of 0.05–0.1, and the corresponding yielding stress, *σ*_y_, is 500 MPa obtained by simulation at the elastic stage. The simulated and experimental unloading curves just achieve a good agreement, when *n* = 0.07, as exhibited in Fig. 2(b). Therefore, the Eq. (3) is obtained as follows:


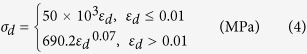


It should be noted that the unit of the stress in the present analysis is MPa. The contour map of the von Mises stress distribution at the maximum depth is displayed in Fig. 2(c). It clearly shows that the stress gradients distribute along the semi-circles, and the red zone, standing for the maximum stress (SMX), is under the indenter. The maximum von Mises stress with a value of 602 MPa, as exhibited in Fig. 2(c), very close to the stress of 604 MPa from Eq. (4), when *ε*_*d*_ is 15%, which indicates the simulation is in good agreement with the experimental results. Figure 2(d) exhibits the stress distribution at the residual depth (DMX) upon unloading. The maximum stress has transfered to the surface, and the stress distribution is irregular. Note that the residual depth (DMX = 1,530 nm) in Fig. 2(d) is in line with the residual depth of 1,550 nm in Fig. 2(b), indicating the accuracy of the current simulation. In a word, *E* = 50 GPa, *σ*_y_ =* *500 MPa, and *n* = 0.07, and the simulated stress-strain relation of the dendrites is available.

### Mechanical properties and microstructures after tension

**Figure 3:**
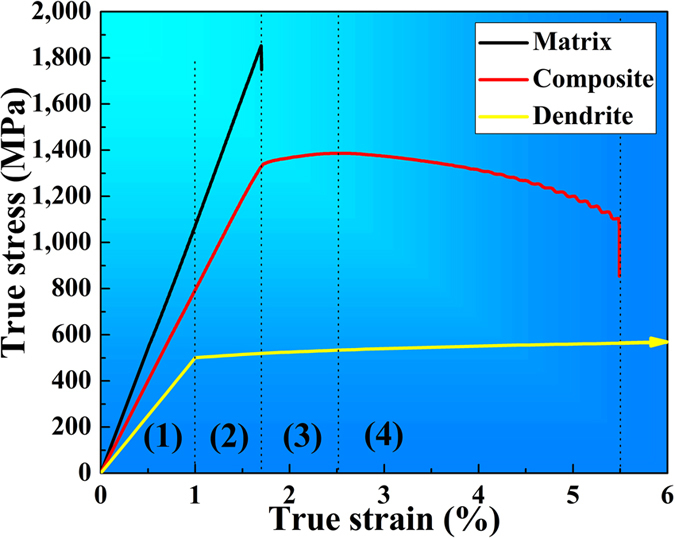
The stress-strain curves of the matrix, the dendrites, and the composites in (**a**). The fractography of the present composites after tension shown in (**b**), the inset in (**c**) indicating the fracture surface.

The pictures of the tensile samples of the present composites and the glass matrix alloy are displayed in the upper and lower portions in Fig. 1(d), respectively. The true stress-strain curves of the present composites and the matrix alloys as well as the dendrites upon tension are displayed in Fig. 3. It should be noted that the stress-strain curve of dendrites is obtained from the combination of the nano-indentation measurements with the FEM analysis. The tensile mechanical properties are summarized in Table 1. The yielding stress, ultrahigh tensile strength, and fracture strain of the present composites are 1,328 MPa, 1,368 MPa and 5.5%, respectively. After yielding, MGMCs exhibit work-hardening behavior until the ultrahigh tensile strength is achieved, and then, softening dominates until the final fracture, similar phenomenon have been widely observed in MGMCs^4^,^5^,^6^,^7^. The matrix phase has a higher yielding stress, *σ*_*ym*_, of 1,853 MPa, but no tension ductility. The elastic strain and yielding strength of dendrites is ~1.0% and 500 MPa, respectively. The fracture strain of dendrites is assumed to be 15%, and the ultrahigh tensile strength is 604 MPa. The Young’s modulus of glass matrix is 103 GPa measured by nano-indentation, which is very closed to the experimental value of 109 GPa from the stress-strain curve in Fig. 3.

**Figure 4:**
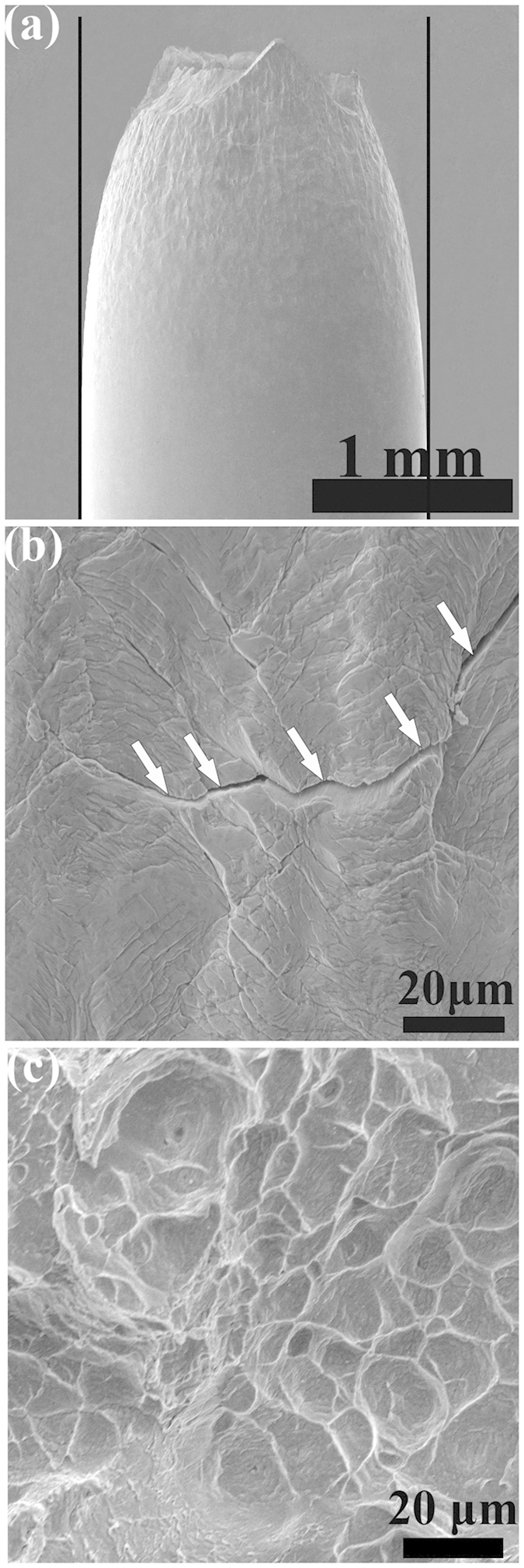
The necking, lateral surface and the fracture surface of the deformed samples after tension subjected to the strain rate of 5 × 10^−4^ s^−1^ shown in (a–c), respectively.

To better understand the deformation mechanisms of *in-situ* MGMCs, it is necessary to analyze the fractographs and microstructure of the present MGMCs after tension. Figure 4(a) shows the lateral surface of the deformed sample after tension. It can be seen that obvious necking is available, which gives an evidence of the distinct tensile ductility. Similar necking associated with softening is widely observed^2^,^4^,^5^,^6^,^7^,^8^,^9^,^10^,^11^,^12^,^15^,^16^. Figure 4(b) shows the magnified SEM image near the fracture surface. Dense patterns of primary and secondary shear bands as well as the microcracks, associated with multi-step shear banding^19^, along the shear bands are visible, indicated by arrows. On the fracture surfaces, two typical features, i.e., some round cores and dimples, are observed, as shown in Fig. 4(c). It is considered that (i) the veins originate from the cores and propagate towards the outside in a radial mode^20^; and (ii) the ridges along dimples reveal the instantaneous increase in temperature rise at the final fracture due to adiabatic heating^21^,^22^.

**Figure 5:**
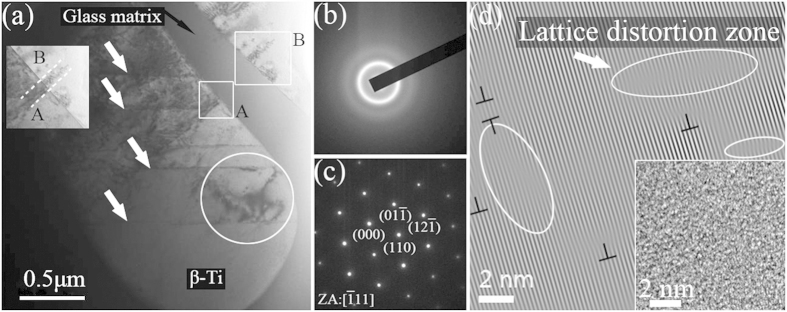
(**a**) TEM bright field image of the deformed composites after tension. The SAED patterns of the glass matrix in (**b**) and the dendrites in (**c**). IFFT images of the dendrites and the glass matrix after tension in (**d**) and inset of (**d**), respectively.

A TEM bright-field image of the deformed MGMCs and selected-area electron-diffraction (SAED) patterns of the amorphous matrix and dendrites are shown in Fig. 5(a–c). The deformed MGMCs contain dendrites in the amorphous matrix, as shown in Fig. 5(a), consisting with that in the as-cast MGMCs, as displayed in Fig. 1(b). The glass matrix can be confirmed by only diffuse halos typical of an amorphous structure, as displayed in Fig. 5(b). The dendrites are identified as body-centered cubic (bcc) phases, i.e., β-Ti dendritic phases. The SAED pattern in Fig. 5(c) obtained from the dendrites corresponds to the 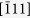 zone axis of the bcc β-Ti solid solution. It can be seen that shear bands pass through the dendrites, indicated by light arrows, and profuse dislocations accumulate within the dendrites forming dislocation tangle, as indicated by light circle in Fig. 5(a). For the dendrites, the deformation structure is clear, as indicated by the inverse fast fourier transform (IFFT) pattern in Fig. 5(d), where a mass of dislocations, denoted by “T”, and lattice distortions, indicated by light ellipses, are present. Similar phenomenon has been found in previous investigation^9^. An very important clue to verify the dendrites dominating in absorbing energy upon deformation is that dislocation tangles initiated by shearing stress terminate within dendrites, as shown in light rectangle B in Fig. 5(a), and the dislocation tangles almost keep invariable when shearing stress pass the glass matrix, as shown in the mosaic graph from zone A and zone B in Fig. 5(a). This phenomenon exhibits that the glass matrix only plays a role in transmitting shearing stress and the microstructures do not change obviously, proved by IFFT pattern of glass matrix in the inset of Fig. 5(d). It is concluded that ductile dendrites dominate in plastic deformation and absorb much plastic energy.

## Discussion

With regard to the current composites, the deformation mechanisms of the glass matrix and crystalline dendrites are coupled upon quasi-static tension. The softening of *in-situ* dendrite-reinforced MGMCs dominates upon tension, and a little work hardening is accompanied^5^,^9^. According to the tensile stress-strain curve of the composites in Fig. 3, the tensile behavior of the *in-situ* dendrite-reinforced MGMCs can be classified into four stages: (1) elastic-elastic, (2) elastic-plastic, (3) plastic-plastic (work-hardening), and (4) plastic-plastic (softening)^9^.

In the elastic-elastic stage, the dendrites and glass matrix are elastic, and the composites are under elastic loading^9^. The stress concentration is caused by the mismatch of Young’s modulli between the dendrites and glass matrix^23^. The stress-strain relations for the matrix is:


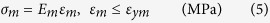


where *σ*_m_, *E*_*m*_, *ε*_*m*_, and *ε*_*ym*_ are the elastic stress, Young’s modulus, elastic strain, and yielding strain of the glass matrix. *E*_*m*_ is equal to 103 GPa measured by nano-indentation tests, which is very close to the experimental value of 109 GPa. The experimental *σ*_*ym*_ is 1,853 MPa. As a consequence, the calculated stress-strain relations of the glass matrix and dendrites at elastic-elastic stage can be expressed as:


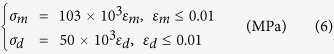


The experimental stress-strain relations of the glass matrix and dendrites are:


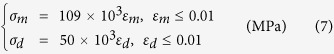


The Young’s modulus of the composites, *E*_*c*_, can be estimated according to Hashin and Shtrikman^24^:


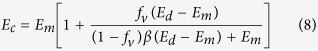


where *f*_*v*_ is the volume fraction of dendrites with a value of 0.42, *β* is the material constant calculated by 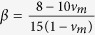, and *v*_*m*_ is the Poisson’s ratio of the glass matrix with a value of about 0.352^25^. Therefore, the *E*_*c*_ = 77 GPa, which is very close to the experimental value of 78 GPa obtained from the stress-strain curve in Fig. 3. The calculated stress-strain relation of the composites can be expressed:


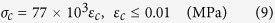


The experimental stress-strain relation of the composites is:


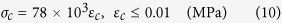


where *σ*_*c*_ and *ε*_*c*_ are the elastic stress and elastic strain of the composites, respectively. With the strain increasing, the dendrites yield first^9^, and the elastic-plastic deformation begins at the second stage.

In the elastic-plastic stage, the stress concentration is increased sufficiently largely to satisfy the yielding criterion of dendrites, i.e. it can lead to the glide of dislocations in the dendrites, and the dendrites deform plastically^9^. In the steady flow, the generation rate of free volumes within glass matrix by the shear-stress driven balances the annihilation rate by the atomic rearrangement^26^, which indicates that the glass matrix still experiences elastic deformation.

According to the Taylor dislocation model^27^, the tensile stress-strain relation of the dendrites is given as:


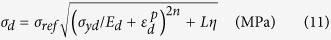


where *σ*_*ref*_is the reference stress of ductile dendrites upon uniaxial tension, and 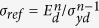, 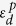 is the plastic strain of the dendrites, and *Lη* stands for the contribution to the work hardening from geometrically necessary dislocations. *L* is the intrinsic material length of the dendrites, and 
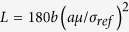
. *μ* and *b* are the shear modulus and Burgers vector of the dendrites, and 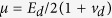. Assume that the Burgers vector of the dendrites, *b*, is about 1 nm^28^. *a* is an empirical material constant in the Taylor dislocation model with a value of 0.3^29^. *η* is the effective plastic-strain gradient, which can be replaced by an average plastic strain gradient, 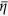. Here, 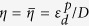, where *D* is the average diameter of the dendrites with a value of 2 μm, as shown in Fig. 1 (b). The equation (11) can be rewritten as:


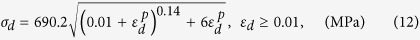


As the dendrites yield, the relationship between the tensile strain of composites, *ε*_*c*_, and that of the dendrites is given by^30^:


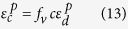


where *c* is the average stress concentration factor of the dendrites, and 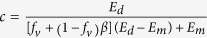
31. 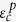 is the plastic strain of composites. The Eq. (13) can be rewritten as 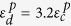. A simple rule of mixture is employed as a first-order approximation to evaluate the axial stress of the composites, *σ*_*c*_:


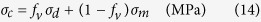


Assuming *ε*_*c*_ = *ε*_*m*_, from Eqs. (6), (12), (13) and (14), the calculated stress-strain relationship at the second stage can be expressed as:


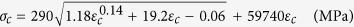



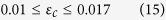


The competition between the creation rates of the shear bands and the multiplication rates of dislocation will determine the mechanical properties of the dual-phase MGMCs^1^. Once the dendrites yield, the plastic misfit between the two phases would lead to a higher stress concentration in the neighborhood at the interface and store a significant elastic energy^23^. As the applied stress is increased continuously, the dislocation density is increased, and numerous dislocations gather at the interface^32^. While the stress concentration is beyond the yielding stress of the glass phase, the shear bands initiate at the interface within the glass matrix^7^,^32^. Microscopically, the local nano-level structure instability leads to the nucleation of shear bands due to a competition between the initiation of shear bands and coalescence of free volumes by a series atomic jump^26^. Both the dendrites and the matrix phase deform plastically, when the tensile stress approaches the yielding stress of the glass matrix. In this case, the dendrites exhibit work hardening, and the shear bands start to initial and propagate in the glass matrix, accompanied by the accommodation of localized plastic deformation^33^.

At the plastic-plastic (work-hardening) stage, by fitting the stress-strain curve of the composites, the constitutive relation can be expressed as follows:


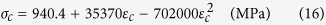


By combining Eqs. (12), (13), (14) and (16), the calculated stress-strain relation of the glass matrix can be described as:


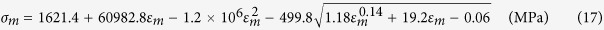


Combining Eqs. (4), (13), (14) and (16), the experimental stress-strain relation of the glass matrix can be expressed as:


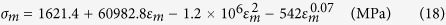


The homogeneous deformation is one of guarantees for use as engineering materials, since inhomogeneous deformation may lead to early failure during service^9^. Therefore, the work-hardening capacity is a precondition for structural applications. The work hardening attributes to the dislocations inside the dendrites, which effectively increases the plasticity of the composites^9^. Additionally, the dendrites were *in-situ* formed during solidification, and the bonding strength of the dendrite-matrix interface is stronger than those made by other methods, i.e., *ex-situ* MGMCs^34^. This strong interface is able to effectively arrest the fast propagation of shear bands, which can be illustrated by the phenomenon that the shear bands change the propagation orientation across the interface^5^,^9^. Hence, the larger stress is needed for the shear bands to propagate in a zig-zag manner resulting in the enhanced plasticity and strength of the composites^9^.

At the plastic-plastic (softening) stage, when many nearly parallel shear bands form and pass through the dendrites collectively, the interfaces cannot deflect the collective movement of the shear bands, the softening occurs, and the samples fail as the shear bands quickly propagate through the whole sample, similar phenomenon has been widely observed^4^,^5^,^6^,^7^,^8^,^9^. Besides, an increase of the free volumes within shear layers will lower the viscosity, which facilitates catastrophic failure^35^. By fitting the stress-strain curve of the composites, the constitutive relation at the fourth stage can be expressed as:


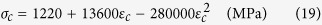


By combining Eqs. (12), (13), (14) and (19), the calculated stress-strain relation of the glass matrix can be described as:


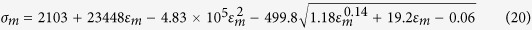


Combining Eqs. (4), (13), (14) and (19), the experimental stress-strain relation of the glass matrix can be expressed as:


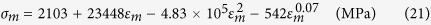


At the plastic deformation stages, the competition between two distinct deformation mechanisms: 1) the damage induced softening of the glass matrix and 2) the work hardening of the dendrites exists all the time^9^. At work-hardening stage, the strain hardening of the dendrites prevails^9^. While, the damage induced softening of the glass matrix dominates at softening stage^9^. The contributions, upon plastic deformation, from work hardening of dendrites and softening of the glass matrix will be equal at a strain of 2.5%:


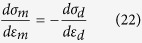


Here, the 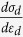 and 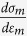 the can be considered as the contributions from the work hardening behavior of the crystalline dendrites and the softening behavior of the glass matrix to the strength of the composites, respectively. At this moment, neither work hardening nor softening is dominating. Later, the composites will experience the strain softening, since shear bands multiply quickly^4^,^5^,^6^,^7^,^8^,^9^. Thus, there are many shear bands on the lateral surface of deformed samples, as shown in Fig. 4(b).

**Figure 6:**
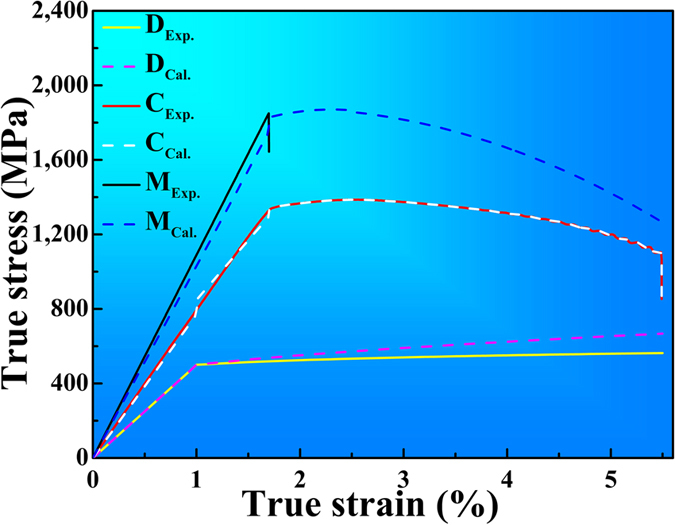
The calculated results vs. experimental results.

As discussed above, the constitutive relations of the dendrites, glass matrix, and composites at four stages have been obtained. These constitutive relations can clarify the corresponding tension behavior. For example, the calculated constitutive relation of dendrites at the plastic stage from Eq. (11), not only reflects the relationship between the stress and strain of dendrites, but also indicates the influence of some key parameters, such as the size of dendritic arms and strain-hardening exponent on the mechanical behavior of the current composites, if combining with the Eq. (14). For instances, the work-hardening capacity of the MGMCs increases with the increase of strain-hardening exponent of dendrites. In this study, the constitutive relations contain two parts: the first one is experimental constitutive relations, and the other is the calculated one. For comparison, the experimental and the calculative stress-strain curves of are drawn in Fig. 6. The solid and dashed lines stand for the experimental and calculated results, respectively. It is concluded that the calculated results are in agreement with the experimental results at each stage, demonstrating the availability of clarification of the tensile behavior by the proposed constitutive relations.

The present models are accurately established based on the tensile behaviors of dendrites, glass matrix, and composites compared to the previous models. The tensile behavior of dendrites is obtained for the first time by combining the nano-indentation measurements and FEM analysis, laying a meaningful foundation to investigate the tensile behavior of *in-situ* dendrite-reinforced metallic glass matrix composites, in addition to opening up a new direction for scientific research. Future work requires finding which parameters greatly influence the work-hardening capacity and tensile ductility of *in-situ* MGMCs so that ductile MGMCs can be designed for engineering applications by tailoring these parameters. Emerging commercialization for these materials includes energy-absorbing structures, biomedical implants, aerospace hardwares, and sporting equipments.

In conclusion, the tensile behavior of *in-situ* dendrite-reinforced metallic glass matrix composites with a composition of Ti_48_Zr_18_V_12_Cu_5_Be_17_ is investigated. The compositions of the glass matrix and dendrites are Ti_33_Zr_19_V_11_Cu_6_Be_31_ and Ti_65_Zr_17_V_14_Cu_4_, respectively. The stress-strain curves of glass matrix and composites are obtained by tension. While, that of dendrites is obtained for the first time by combining the nano-indentation measurements and FEM analysis, laying a meaningful foundation to investigate the tensile behavior of *in-situ* dendrite-reinforced metallic glass matrix composites. Based on the stress-strain curve of the composites, the tensile behavior of the present *in-situ* MGMCs can be classified into four stages: (1) elastic-elastic, (2) elastic-plastic, (3) plastic-plastic (work-hardening), and (4) plastic-plastic (softening). The constitutive relations at each stage are established, and the calculated results and experimental results are in good agreement, giving an obvious clue to clarify and predict the tensile behavior of such kind of *in-situ* dendrite-reinforced metallic glass matrix composites. The TEM results display that ductile dendrites could absorb much plastic energy during the plastic deformation.

## Methods

The *in-situ* MGMCs with a composition of Ti_48_Zr_18_V_12_Cu_5_Be_17_ (at. %) were prepared by arc melting and were cast into a copper mold. The dimension of the cast ingots was 6 × 80 mm^2^ (diameter × length). The phases of the cast ingots were characterized by X-ray diffraction (XRD) with Cu *K*_*α*_ radiation. The microstructures and chemical compositions were examined by scanning-electronic microscope (SEM), equipped with energy-disperse spectrometer (EDS). The matrix alloys were prepared by arc melting and were cast into a copper mold, and the dimension of the cast ingots was 1.5 × 4 × 50 mm^3^ (thickness × width × length). The tensile samples of the MGMCs and the glass matrix alloys with the gauge dimensions of 2 × 15 mm^2^ (diameter × length) and 1.5 × 4 × 8 mm^3^ (thickness × width × length), respectively, were prepared. The mechanical properties of BMGs are in good agreement in bulk scale, regardless of the dimension of the bulk cast ingots^36^,^37^,^38^. The quasi-static tensile tests were conducted at room temperature at a constant strain rate of 5 × 10^−4^ s^−1^. Each test was repeated for at least 5 times. Finally, the deformed samples were observed by SEM, transmission-electron microscopy (TEM; JEM 2010F; Tokyo, Japan), and high resolution transmission electron microscopy (HRTEM) to analyze the deformation mechanisms.

In order to obtain the stress-strain curve of the dendrites, a method, which combines the finite-element-method (FEM) analysis and the nano-indentation experiments, was employed here. A Nano Indenter II tester (MTS Systems, USA) with a trihedral Berkovich indenter was used to calculate the Young’s modulus of both the glass matrix and dendrites at room temperature. Thermal drift correction of the machine was kept below 0.05 nm/s during each test, and the loading holding time was settled as 10 s. Indentations for the Ti_48_Zr_18_V_12_Cu_5_Be_17_ sample at a strain rate of 0.05 s^−1^, within a 2000 nm depth limit were adopted to evaluate the elastic moduli of the dendrite phase and matrix phase.

## Additional Information

**How to cite this article**: Sun, X.H. *et al.* An improved tensile deformation model for *in-situ* dendrite/metallic glass matrix composites. *Sci. Rep.* **5**, 13964; doi: 10.1038/srep13964 (2015).

## Figures and Tables

**Table 1:** The different mechanical parameters of the composites, the matrix, and the dendrites.

Alloys	Yield strength (MPa)	Tensile strength (MPa)	Young’s modulus (GPa)	Fracture strain (%)	Elastic strain (%)
Composites	1,328	1,386	78	5.5	1.7
Matrix	1,853	1,853	109	1.7	1.7
β dendrites	500	604	50	15	1.0
